# A new bioluminescence-based tool for modulating target proteins in live cells

**DOI:** 10.1038/s41598-019-54712-y

**Published:** 2019-12-03

**Authors:** Tetsuya Ishimoto, Hisashi Mori

**Affiliations:** Department of Molecular Neuroscience, Graduate School of Medicine and Pharmaceutical Sciences, University of Toyama, Toyama, Japan

**Keywords:** Cytological techniques, Actin

## Abstract

We have developed a new genetically encoded tool designed to generate reactive oxygen species (ROS) at target proteins in cultured cells; it is designed using firefly luciferase and photosensitiser protein KillerRed. Targeting this fusion protein, KillerFirefly, to F-actin in live cells and treatment with luciferin induced a characteristic structure, previously reported as a cofilin-actin rod, which is seen in patients with Alzheimer’s disease. This structural change is considered to be elicited by the consistent generation of very low-level ROS by KillerFirefly in the vicinity of F-actin. Moreover, our results suggest the presence of an actin-regulating system, controlled by very low levels of endogenously generated ROS.

## Introduction

Methods of inactivating particular protein functions, in living cells or animal subjects, are important for fundamental biological and applied biomedical research. Several gene-based inactivation methods, such as RNAi^1^ and genome editing^2^, have been developed and considered for medical purposes. Similarly, protein-based inactivation approaches, such as chromophore assisted laser inactivation (CALI), have also been developed^3^. CALI is an advanced technique that allows the spatiotemporal control of molecular inactivation by subcellular ROS generation. However, laser toxicity is known to cause problems, making long-term laser usage difficult; more significantly, it is difficult to uniformly irradiate wide areas of cultured cells or whole animals using lasers.

In this study, we employed firefly luciferase and the photosensitiser protein KillerRed to overcome these disadvantages of conventional CALI. Firefly luciferase is a light-emitting protein that is activated by its substrate luciferin (emission maximum of 560 nm)^4^. The luciferase-luciferin reaction has been used to monitor transcription in cultured cells, with good signal-to-noise ratio and signal linearity^5^. Furthermore, the expression of luciferase in animals with a promoter for a particular gene enables the use of *in vivo* imaging when luciferin is injected intraperitoneally^6,7^. KillerRed is a photosensitising fluorescent protein that was developed by mutation of hydrozoan chromoprotein anm2CP^8,9^. This protein produces O_2_^−^ in response to light irradiation (maximum excitation at 585 nm) and has been used in the CALI technique. Inactivation of many cellular proteins and functions using KillerRed has been reported^10–13^.

**Figure 1:**
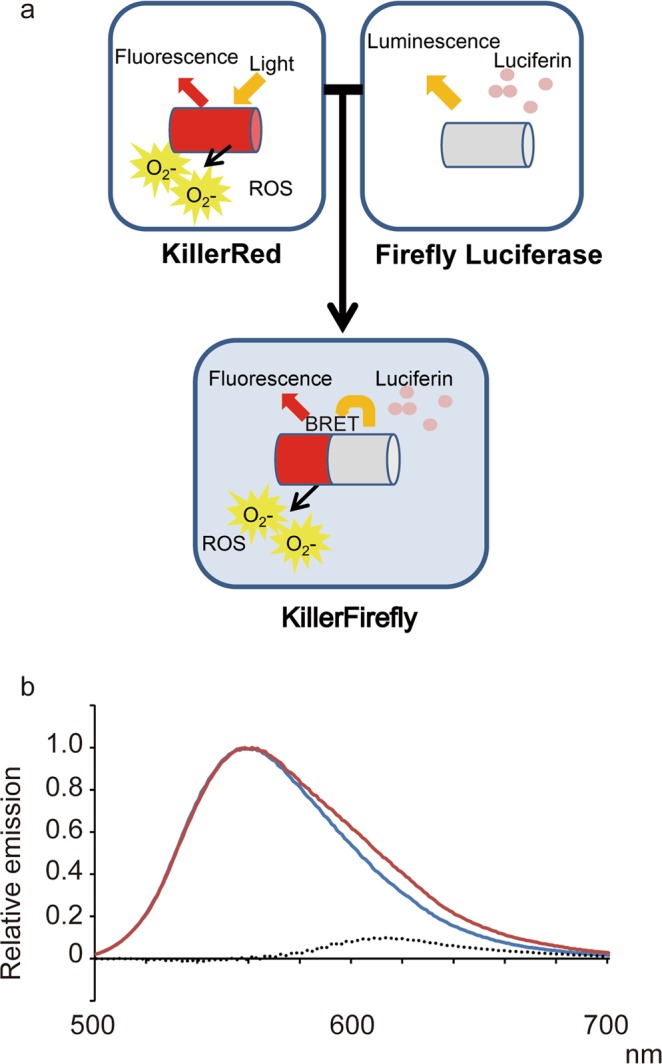
Development of the KillerFirefly protein. (**a**) Principal of the technique described in this report. (Upper) KillerRed protein is a fluorescent protein which generates ROS when excited by yellow light. Firefly luciferase emits light (maximum at 560 nm) depending on luciferin, a substrate molecule. (Lower) Fusion protein named KillerFirefly consists of KillerRed and firefly luciferase, which generates ROS via bioluminescent resonance energy transfer (BRET) from luciferase. (**b**) Spectrum analysis of the light emitted by KillerFirefly and luciferase. Red and blue lines represent the spectrum of KillerFirefly and firefly luciferase, respectively. Dotted line indicates subtracted value from spectrum of KillerFirefly by that of luciferase.

We constructed a fusion protein of KillerRed and firefly luciferase (KillerFirefly, Fig. 1a), which was expected to generate ROS from KillerRed when excited by the bioluminescence resonance energy transfer (BRET) by luciferase in response to the luciferin treatment. We evaluated whether targeted ROS generation by the KillerFirefly protein modifies the function of cellular protein.

## Results

### Establishment of KillerFirefly protein that emits ROS in response to luciferin treatment

KillerRed and firefly luciferase were fused (KillerFirefly) and successfully expressed in HEK293T cells. To test whether KillerRed is excited by luciferase via the BRET effect, the spectrum of emitted light from KillerFirefly was measured and compared with that of luciferase (Fig. 1b). The subtracted spectrum (dotted line) peaked at 610 nm, which was the reported emission maximum of KillerRed^8^. Increase in BRET ratio (emission at 610 nm/emission at 560 nm) was 1.23. This result indicates that KillerRed is excited by BRET from luciferase. Because excitation of the KillerRed protein evokes ROS generation^8^, we concluded that the KillerFirefly protein generates ROS in response to luciferin treatment in live cells. However, quantification of generated ROS using conventional nitro blue tetrazolium (NBT) and NIR-CLA methods was not successful, probably because the amount of ROS was insufficient.

**Figure 2:**
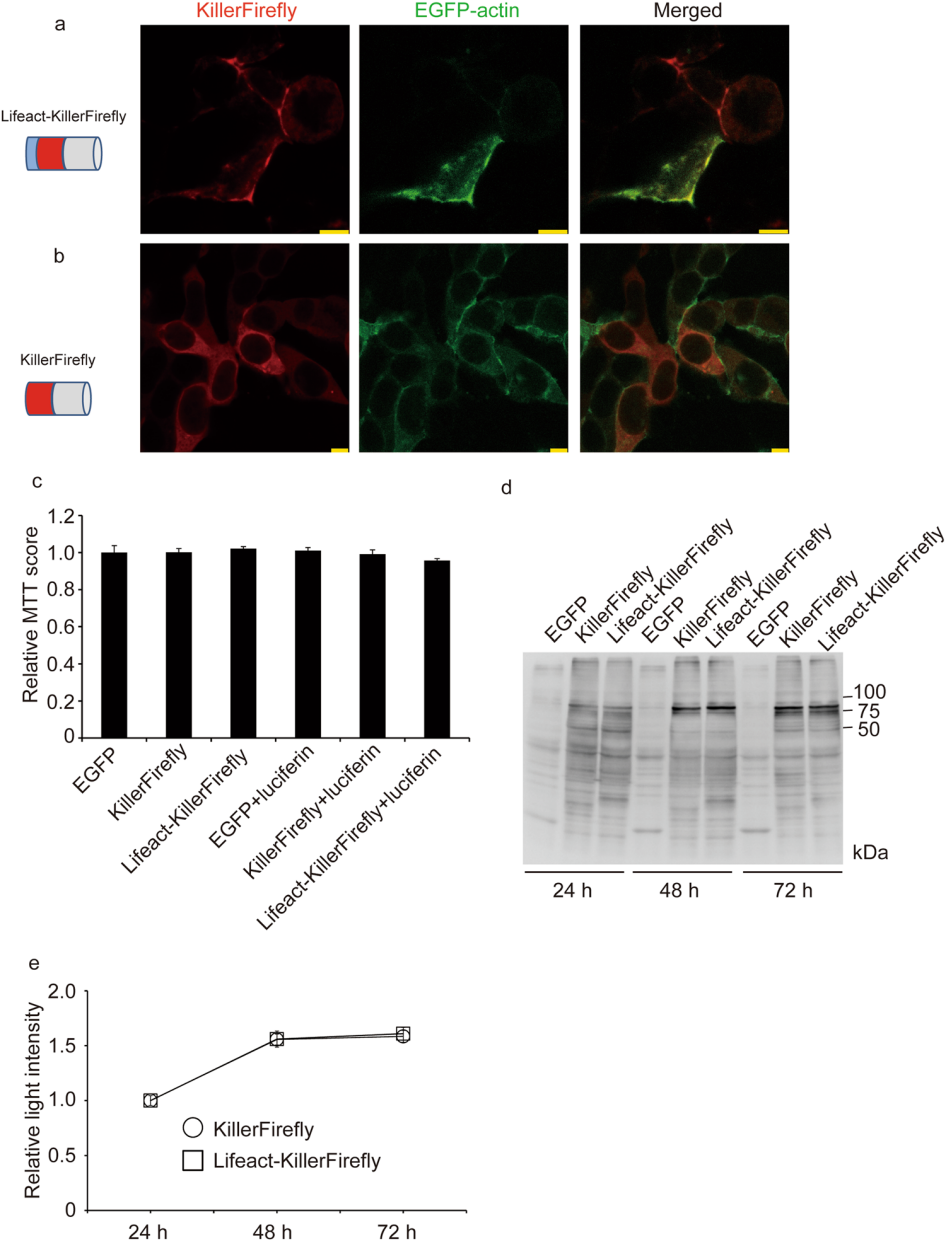
Subcellular localisation of KillerFirefly protein. (**a**) EGFP-actin (Green) and Lifeact-KillerFirefly (Red) expression in HEK293T cells. (**b**) EGFP-actin (Green) and KillerFirefly (Red) expression. Bars indicate 7.5 μm. (**c**) Effect of KillerFirefly and Lifeact-KillerFirefly expression on HEK293T viability assayed by MTT method. Relative MTT score (mean ± SEM, n = 4) to EGFP expressing HEK293T two days after transfection is represented. (**d**) Expression of Lifeact-KillerFirefly in HEK293T cells at indicated time point after transfection analysed by western blotting using anti-luciferase antibody. A chemiluminescent image of single full length blot without any brightness and contrast alteration is shown. The locations of molecular weight markers (kDa) are shown on the right. (**e**) Relative intensity (mean ± SEM, n = 4) of light emission from Lifeact-KillerFirefly (□) and KillerFirefly (○) expressing HEK293T at indicated time point after transfection.

Further, we attempted to target the KillerFirefly protein to F-actin, to investigate the effect of targeted exposure of ROS on actin polymerisation. Actin is a cytoskeletal protein, for which polymerisation and depolymerisation are crucial for many cellular functions, such as migration^14^, cancer cell invasion^15^, synaptic plasticity^16^, and cell death^17^. Lifeact is an F-actin-binding peptide consisting of 17 N-terminal amino acids of ABP120 protein^18^, and Lifeact fused with fluorescent protein has been used for F-actin imaging in live cells^19^. HEK293T was transfected with EGFP-actin and Lifeact-KillerFirefly and subcellular localisation of the transfected proteins was analysed using confocal microscopy. We found these two fusion proteins colocalised to the periphery of cells (Fig. 2a). However, KillerFirefly protein was present uniformly throughout the cell body (Fig. 2b). This result demonstrates that KillerFirefly successfully targeted F-actin via the Lifeact peptide, and KillerFirefly was not enriched in any subcellular organelles. Then we tested whether Lifeact-KillerFirefly expression was toxic to HEK293T cells. Three days after plasmids transfection with or without luciferin, cell viability was measured using 3-(4,5-dimethyl-2-thizolyl)-2,5-diphenyl-2*H*-tetrazolium bromide (MTT) assay. No significant decrease in relative MTT score in KillerFirefly and Lifeact-KillerFirefly expressing cells to EGFP expressing cells was detected, which indicated no toxicity was induced by KillerFirefly and Lifeact-KillerFirefly expression with or without luciferin (Fig. 2c). Physical stability of Lifeact-KillerFirefly was analysed using western blotting and found that full length Lifeact-KillerFirefly (86 kDa) was present three days after transfection, which means Lifeact-KillerFirefly is stable in HEK293T cells. However, expressed protein seemed to be partially degraded (Fig. 2d). Decrease in light intensity from KillerFirefly and Lifeact-KillerFirefly was not observed three days after transfection, which indicates light emitting activity of Lifeact-KillerFirefly is kept at least three days after transfection (Fig. 2e).

### Rod-like structure of actin induced by low-level ROS generated by Lifeact-KillerFirefly

**Figure 3:**
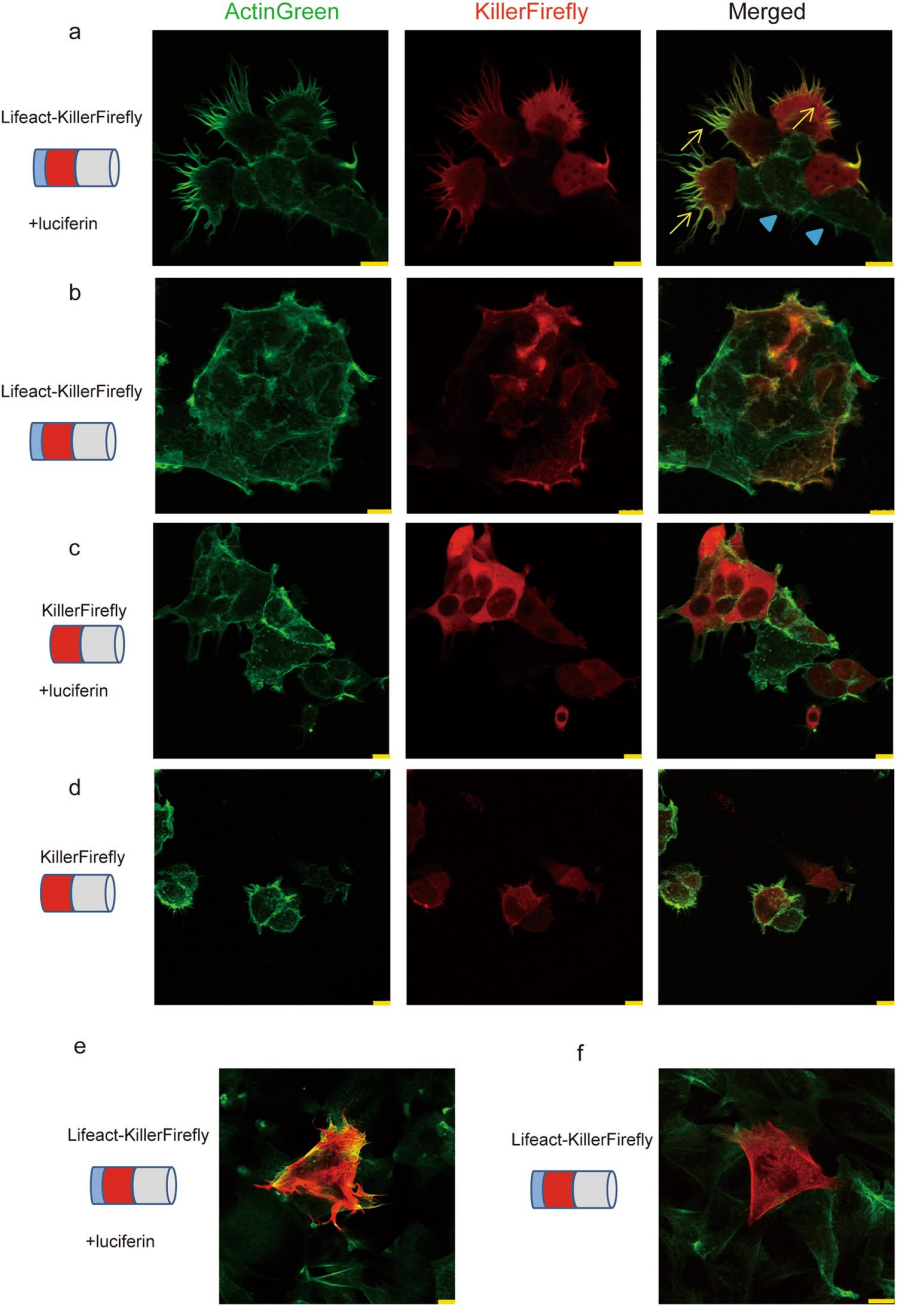
Structural modulation of F-actin by Lifeact-KillerFirefly. (**a**,**b**) Lifeact-KillerFirefly (Red)-expressing HEK293T cells, with or without luciferin (2 mM, 24 h) treatment, were stained with ActinGreen. Rod-like structures of F-actin were seen only in cells expressing Lifeact-KillerFirefly with luciferin (arrows), not in Lifeact-KillerFirefly-absent cells (arrowheads). (**c**,**d**) Cells expressing KillerFirefly did not show any rod-like structures. (**e**,**f**) Lifeact-KillerFirefly (Red)-expressing CHO cells, with or without luciferin (2 mM, 24 h) treatment, were stained with ActinGreen. Bars indicate 10 μm.

To determine the effect of targeted ROS generation in the vicinity of F-actin, luciferin (2 mM, 24 h) was added to the culture medium of Lifeact-KillerFirefly-expressing cells. Many of the luciferin-treated cells possessed filopodia-like protrusions that were F-actin-positive (Fig. 3a arrows). Cells without the expression of Lifeact-KillerFirefly did not show such rod-like structure (Fig. 3a arrowheads). In the control experiment, HEK293T cells that expressed Lifeact-KillerFirefly without luciferin, and KillerFirefly protein with or without luciferin, did not show rod-like structures (Fig. 3b–d). In addition to HEK293T cell, we observed increased number of protrusions which was F-actin positive induced by Lifeact-KillerFirefly and luciferin treatment in CHO cells (Fig. 3e,f). This result suggests that effect of KillerFirefly on actin polymerisation is common to many cell types.

**Figure 4:**
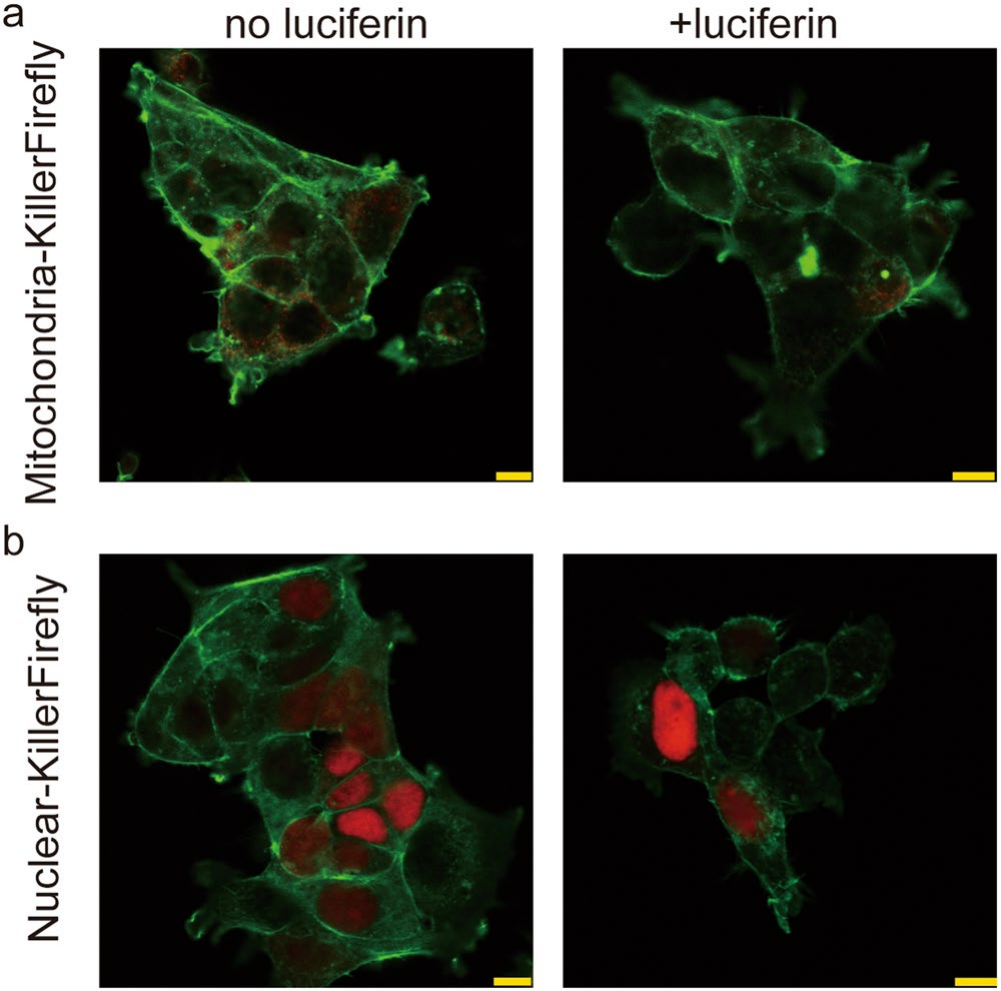
Effect of nuclear and mitochondrial-targeted KillerFirefly on actin structure. (**a**) Mitochondrial-KillerFirefly and (**b**) Nuclear-KillerFirefly were expressed and treated with luciferin (2 mM, 24 h). The red signal represents fluorescence of KillerFirefly. The green signal represents fluorescence of ActinGreen. Bars indicate 10 μm.

Next, we constructed other fusion proteins in which nuclear- and mitochondrial- localisation peptides were fused to the N-terminal of KillerFirefly. Those proteins were expressed in HEK293T and treated with luciferin and successfully localised to the nucleus and mitochondria; however, the rod-like F-actin structure was not observed (Fig. 4a,b). This result implies ROS only induces actin reorganisation when generated close to F-actin.

### Increased actin polymerisation elicited by activation of Lifeact-KillerFirefly

**Figure 5:**
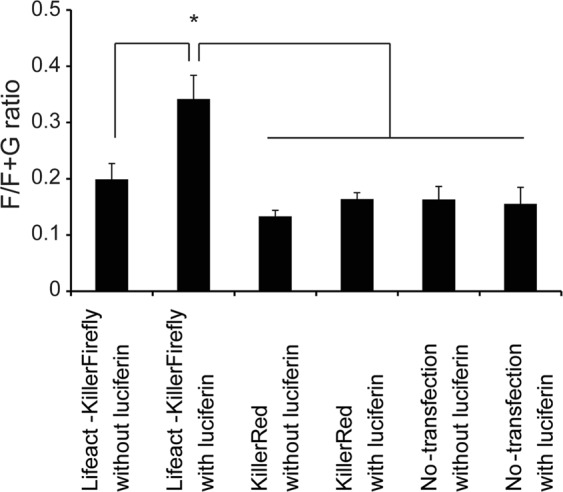
Lifeact-KillerFirefly-induced change of F- and G-actin ratio. Ratio of F/F + G-actin was measured and quantified by western blotting after separation of F- and G-actin using ultracentrifugation. Vertical axis represents F/F + G-actin ratio of total actin. Bars indicate mean ± SEM (*P < 0.05, n = 5, Student’s t-test).

We determined whether the F- and G-actin ratio changed in cells expressing Lifeact-KillerFirefly and treated with luciferin, since the result in Fig. 3a seemed to suggest targeted ROS generation increased F-actin content. F- and G-actin in the luciferin-treated cells were separated by ultracentrifugation and analysed by western blotting, to examine whether F-actin content was up-regulated in Lifeact-KillerFirefly-expressing cells after luciferin treatment. We found the F/F + G-actin ratio increased in luciferin-treated Lifeact-KillerFirefly-expressing cells (Fig. 5), indicating that the rod-like structures consist of newly polymerised actin.

### Colocalisation of cofilin and F-actin induced by Lifeact-KillerFirefly

**Figure 6:**
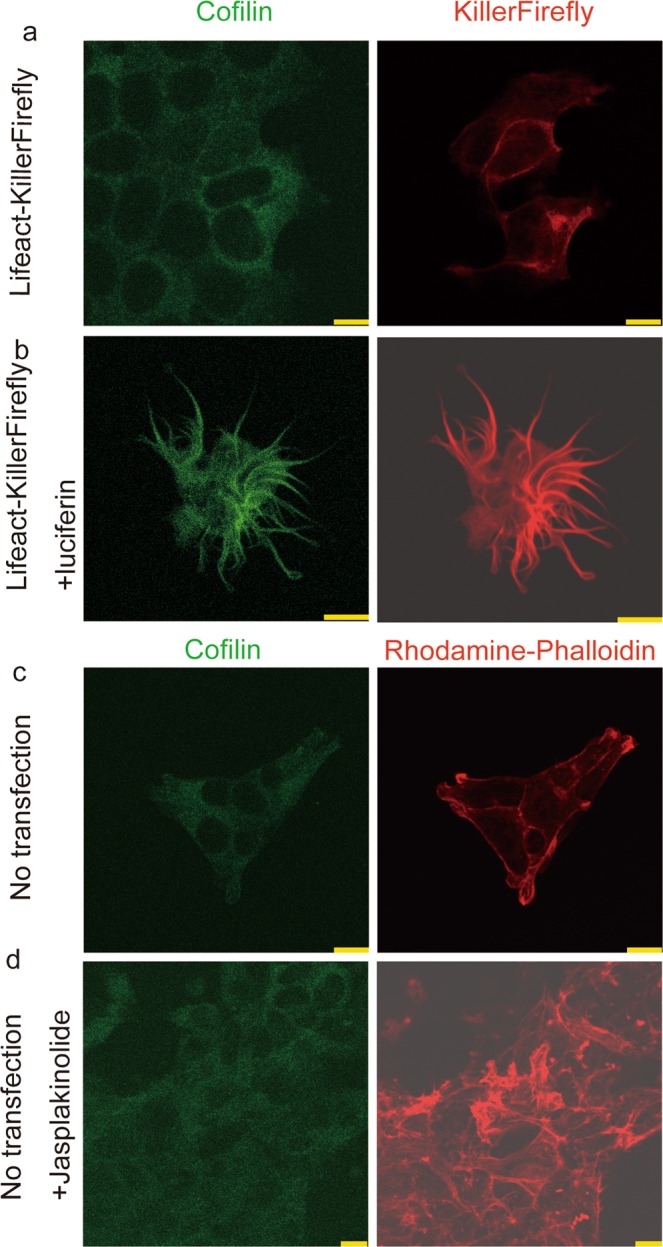
Colocalisation of cofilin and structurally modulated F-actin, induced by Lifeact-KillerFirefly. (**a**,**b**) Localisation of cofilin in Lifeact-KillerFirefly-expressing cells. Green and red signals represent cofilin immunoreactivity and Lifeact-KillerFirefly, respectively. (**c**,**d**) Cofilin and F-actin staining after jasplakinolide treatment. Green and red signals represent cofilin and rhodamine-phalloidin, respectively. Bars indicate 7.5 μm.

There are known to be several types of F-actin structure that differ in length, bundling, and binding proteins^20^. We, therefore, tried to elucidate which type of F-actin structure was induced by F-actin-targeted ROS generation. Luciferin-treated Lifeact-KillerFirefly-expressing cells were fixed and immunostained with anti-cofilin antibody. We found that cofilin accumulated in the structures induced by Lifeact-KillerFirefly and luciferin (Fig. 6b). Since Lifeact-KillerFirefly and F-actin colocalised (Fig. 2a), cofilin and F-actin must also have colocalised. However, the cofilin signals in non-luciferin-treated cells were uniformly diffused through the cytoplasm, even though the Lifeact-KillerFirefly signal was enriched in the cell peripheries (Fig. 6a).

We then analysed cofilin localisation in the cells treated by the F-actin-stabilising reagent jasplakinolide^21^, to test whether up-regulation of F-actin was sufficient to induce cofilin accumulation. However, we detected no cofilin accumulation even when F-actin was up-regulated by jasplakinolide (Fig. 6c,d). These results indicate F-actin-cofilin colocalisation is not solely induced by increased F-actin but by the targeted generation of ROS close to F-actin.

## Discussion

We have developed a new technique for generating ROS in the vicinity of subcellular targets, such as F-actin, by employing KillerRed, firefly luciferase, and a localisation peptide (Lifeact). In this technique, adding luciferin (a substrate of firefly luciferase) is sufficient to generate ROS at subcellular targets. Using luciferin instead of a laser, as in conventional CALI, conveys several advantages. Unlike lasers, luciferin is not toxic to the cells, so long-term luciferin treatment followed by long-term ROS generation in the cultured cells is feasible. Furthermore, all the cultured cells in a dish can be uniformly treated by luciferin, so researchers can perform biochemical analysis, such as western blotting, on cell lysate after ROS generation (Fig. 5). In addition, the amount of ROS generated can be altered by changing the luciferin concentration.

We found that the F-actin structure induced by targeted ROS generation colocalised with cofilin (Fig. 6). Cofilin is an actin-binding protein that forms a rod-like structure with actin called a cofilin-actin rod. Cofilin-actin rods are reported to be formed by ROS generation in the cell^22^, are seen in the brain of Alzheimer’s disease patients^23,24^, and are thought to cause the neuronal dysfunction^25^ underlying cognitive impairment. Therefore, it is reasonable to assume that cofilin-actin rods were induced by F-actin-targeted ROS generation by Lifeact-KillerFirefly. Transgenic mice expressing Lifeact-KillerFirefly in neurons may serve as models of Alzheimer’s disease to explore the role of cofilin-actin rods on cognitive dysfunction.

In this study, we could not measure the amount of ROS generated by KillerFirefly by conventional ROS measurement methods (data not shown), which is likely to be due to the low levels of ROS generated by KillerFirefly; this may be because the light intensity of luciferase is considerably weaker than that of CALI lasers. However, our results indicate that long-term ROS exposure, even at very low levels, is sufficient to alter actin organisation.

We found that mitochondrial and nuclear localisation of KillerFirefly did not induce change in the F-actin structure after luciferin treatment (Fig. 4), while Lifeact-KillerFirefly did (Fig. 3a). These results indicate that generated ROS is not diffusible and oxidises only the proteins adjacent to KillerFirefly. The oxidisation of target proteins, while other proteins are unaffected, is a major advantage of our method. Since bath-application of hydrogen peroxide to the culture medium does not always form cofilin-actin rods^26,27^, targeted generation of ROS should be considered very different. The fact that very small amount of local ROS, below the detection limit, can induce dynamic changes in F-actin structure, suggests that cellular actin is also physiologically modulated by endogenously generated low-level local ROS. The candidate physiological superoxide generators are NADPH oxidases (NOXs), which are known to show specific subcellular localisation depending on the isoform^28^, for example, NOX2 is demonstrated to be present at synapses^29^. KillerFirefly may be used to mimic NOXs, to analyse the relationships between neuronal function, the cytoskeleton, and local ROS generation. This method could be used to modulate other proteins, if they can be targeted by specific binding peptides, such as Lifeact. Luciferin, once injected intraperitoneally, diffuses throughout the bodies of animals^6^; therefore, *in vivo* experiments using transgenic, KillerFirefly protein-expressing, mice may be possible.

## Methods

### Spectrum measurement

KillerFirefly-expressing HEK293T was harvested and homogenised using BioMasher (Nippi) in a Tris-buffer (100 mM Tris-Hcl pH 8.0). Lifeact-KillerFirefly protein was transferred to a 96-well plate (Nunclon Delta Surface, Thermo Fisher) and luciferin (1 mM final) was added to each well. Spectrum data was collated using SpectraMax i3 (Molecular Devices).

### Plasmids

A fragment of firefly luciferase (luc2, Promega) was added to the C-terminus of KillerRed expressing vector (pKillerRed-N, Evrogen), using conventional molecular biological techniques. We named this fusion protein ‘KillerFirefly’, and the subcellular localisation peptides (Lifeact: MGVADLIKKFESISKEE; nuclear localisation peptide: MDPKKKRKVDPKKKRKV; and mitochondria localisation peptide: tandem sequence of MSVLTPLLLRGLTGSARRLPVPRAKIHSLPPEGKL) were added to the N-terminal of the KillerFirefly protein to allow analysis of the effect of local ROS generation.

### ROS measurement

For the NBT method, KillerFirefly-expressing HEK293T was treated with 2 mM luciferin and 1 mg/ml of NBT for 1 h in a CO_2_ incubator. The precipitate was dissolved in DMSO and absorbance at 560 nm was measured^30^. For the NIR-CLA method, KillerFirefly- expressing HEK293T was harvested and homogenised using BioMasher (Nippi) in PBS and treated with 2 mM luciferin and 10 µM NIR-CLA (Atto). Luminescence was measured using an Aequoria-2D/C8600 system (Hamamatsu photonics).

### Western blotting

Proteins from HEK293T were separated by SDS-PAGE and transferred to a PVDF membrane (Millipore) by electroblotting. After the membranes were incubated sequentially with anti-actin (Santa Cruz, 1:2000) or anti-luciferase antibody (Promega, 1:500), followed by HRP-conjugated secondary antibodies, a signal was developed by Luminata Forte Western HRP Substrate (Merck Millipore), and detected by LAS-4000 mini system (GE Healthcare).

### Cell culture and transfection

HEK293T and CHO cells were cultured in DMEM (Nacalai) supplemented with 10% FBS, using Glass-base dish (Iwaki) at 37 °C in 5% CO_2_. Transfection to HEK293T and CHO was performed using Lipofectamin 3000 (Thermo Fisher) and Trans-it CHO (Mirus), respectively. Luciferin was added to the culture medium 24 h after transfection and cells were cultured for 24 h.

### Cell viability assay

Cell viability was determined by MTT method. Cells were incubated with the MTT (Nacalai, 1 mg/ml) for 2 h in the CO_2_ incubator. Deposited formazan was solubilised with 1 ml of dimethyl sulfoxide, and the absorbance at wavelengths of 570 and 630 nm were measured using a spectrophotometer (Gene Quant 1300, GE Healthcare). Sample signal intensity was obtained by subtraction of OD at 630 nm from OD at 570 nm and indicated as relative value to the control.

### Cytochemical staining and image acquisition

HEK293T cells were fixed with 4% paraformaldehyde in PBS and washed twice with PBS. KillerFirefly was detected using red fluorescence emitted by its component KillerRed. For F-actin staining, cells were then reacted with ActinGreen 488 ReadyProbes reagent (Thermo Fisher), according to manufacturer’s instruction. Briefly, the reagent was diluted 15 times in PBS and incubated with fixed cells for 30 min. Then the staining solution was replaced by PBS. For F-actin staining in Fig. 6, cells with or without jasplakinolide treatment (200 nM, for 2 h) were fixed and incubated with 0.14 μM rhodamine-phalloidin/PBS for 1 h at room temperature and washed with PBS. For cofilin immunocytochemistry, fixed cells were incubated with an anti-cofilin antibody (Abcam, 1:100) at 4 °C overnight in 1% FBS supplemented PBS. Then cells were washed and incubated with Alexa488 (Thermo Fisher)-conjugated secondary antibody at room temperature. Fluorescence images were acquired using a laser confocal microscopy system (TCS-SP5, Leica). Argon and green diode laser were used to acquire green and red fluorescence, respectively.

### F/G-actin separation of cell lysate by ultracentrifugation

A G-actin/F-actin *in vivo* assay kit (Cytoskeleton) was used to separate F- and G-actin from the HEK293T lysate. HEK293T cells were lysed in the cell lysis and F-actin stabilisation buffer that was provided with the kit. The cell lysate was centrifuged (Optima TLX, Beckman) at 100,000 × g for 1 h at room temperature. The pellet and supernatant were collected and labeled as the F-actin-and G-actin-containing fractions, respectively. The amount of F-actin and G-actin were detected with western blotting as described above.

### Statistics

Data were analysed using a two-tailed Student’s t-test (Figs. 2c,e and 5). Values in graphs are expressed as mean ± SEM. The significance level was 0.05.
